# The user experience of ambulatory assessment and mood monitoring in depression: a systematic review & meta-synthesis

**DOI:** 10.1038/s41746-025-02118-8

**Published:** 2025-12-02

**Authors:** Laurence Astill Wright, Madiha Majid, Georgina Shajan, Goldie Momoh, Renee Patil, Mat Rawsthorne, Daljit Purewal, Shireen Patel, Richard Morriss

**Affiliations:** 1 Institute of Mental Health, University of Nottingham, Nottingham, UK; 2 Centre for Academic Mental Health, Population Health Sciences, University of Bristol, Bristol, UK; 3 Coventry and Warwickshire Partnership NHS Trust, Coventry, UK; 4 Virtual Health Labs Ltd, Banbury, UK; 5 NIHR ARC East Midlands, University of Nottingham, Nottingham, UK; 6 Nottingham NIHR Biomedical Research Centre, University of Nottingham, Nottingham, UK; 7 NIHR MindTech Medical Technology Collaborative, University of Nottingham, Nottingham, UK

**Keywords:** Psychiatric disorders, Depression

## Abstract

The preferences and opinions of individuals with depression will likely be fundamental for the success of mood monitoring interventions, or for ambulatory assessment approaches as methods of data collection. Concerns have been raised regarding negative psychological effects of repeated mood assessment. This systematic review and meta-synthesis of qualitative studies assessed the user experience of mood monitoring and ambulatory assessment procedures. This included: barriers and facilitators to use for people with depression and for clinicians, negative psychological effects and the intended purpose of use. Eight electronic databases were searched and mixed-methods studies were included. Qualitative studies were rated for risk of bias. Fourteen studies were identified. We identified seven overarching concepts: negative psychological effects, perceived effectiveness, difficulties in completing questionnaires, sharing with others, desired features, purpose of mood monitoring, and clinician barriers/facilitators. While many participants found the mood monitoring/ambulatory assessment therapeutic and positive, many participants reported negative consequences from ambulatory assessment/mood monitoring. Future protocols should monitor negative psychological effects, whether they are long-lasting and consider testing the incorporation of additional therapeutic elements to manage them. We report additional key concepts that are likely to improve the user experience, engagement, attrition, usability and acceptability of ambulatory assessment/mood monitoring protocols for people with depression.

## Introduction

This review assesses the user experience of mood monitoring, mood tracking and ambulatory assessment procedures in people with unipolar depression. Ambulatory assessment is a broad group of methods that uses mobile technology to study people repeatedly, often in natural settings and real time^1^. Ambulatory assessment includes remote measuring technology (e.g. wearables to collect data in the background, or passively), mood monitoring and ecological momentary assessment (EMA—more intensive reporting of data at random e.g. multiple times per day in the participant’s environment^2–4^). Developments in digital technology, such as smartphones and wearable devices have led to the generation of new digital mood monitoring tools designed for people with depression^5^. There are two different types of monitoring tools. One uses active monitoring and require users to actively input data via self-report questionnaires. Others collect data via smartphone sensors or wearables and this is termed passive data^6^. This study examines the user experience in mood monitoring studies and some of these studies are also remote measuring technology, ambulatory assessment and EMA studies and there is definitional overlap. Mood monitoring can be used as an intervention and also as a method of ascertaining outcome. Both of these purposes are included and discussed here.

These electronic tools have many promising potential use cases. Self-monitoring interventions offer people with depression a potential method of self-managing their symptoms^5^. This may be facilitated by improved insight and subsequent behaviour change via the detection of early warning signs of relapse into depression or worsening of low mood^7^. The tools could also help ascertain clinical objectives for treatment in collaboration with clinicians, or be used as research tools to identify digital depressive phenotypes to potentially improve treatment personalisation in mental health care. These novel methods of active and passive data collection may improve on existing research methods by decreasing participant burden and allowing for a higher quality and granularity of mood assessment at decreased economic cost. This may improve the efficiency of conducting large studies with higher quality data potentially allowing the assessment of more personalised approaches. In many healthcare systems access to high-intensity psychological therapy or specialist mental health care is limited and improving access to empirically supported therapies for people with depression is a significant global challenge^8^. Digital approaches offer a potential solution to some of these problems. Considering the significant potential of new measurement technologies, we have a limited understanding of the acceptability, feasibility and validity of mood monitoring and ambulatory assessment approaches in clinical mental health care or research^9^.

To ensure mood monitoring interventions meet the needs of people with depression their perspectives are likely to be fundamental to its success^10^. Likewise, to maximise participation in research-based ambulatory assessment, the procedure must be usable and acceptable to people with depression^10^. If ambulatory assessment is acceptable/feasible for some populations but not for others, there is a risk of widening health inequalities. Ambulatory assessment protocols for research are likely to require considerable commitment from the user, and any usability issues may have subsequent implications for the integrity of the research^11^. As some of these procedures can include multiple components, it is possible that there might be potential benefits and challenges to the inclusion of specific components^12^. Modification of these may improve the acceptability of the intervention/ambulatory assessment to people with depression^12^.

In this review, we refer to the use of a mood monitoring/ambulatory assessment protocol as the particular use of mood monitoring/ambulatory assessment described in the included paper and the actions that follow it. This could refer to a research protocol, or to the intervention and the procedures that follow as consequences of using mood monitoring/ambulatory assessment in a particular way. The protocol does not refer to the quality of the methodology or reporting of the study. Research exploring how people with depression experience ambulatory assessment and mood monitoring is lacking, particularly in long-term real-world use^13,14^. Many of these studies have been limited by small sample sizes in short-term use, hypothetical scenarios or just examining one specific mood monitoring protocol^14^. Furthermore, people with depression seem to use these tools in a variety of different ways and an understanding of this is key to the future implementation of these approaches^15^. Fundamental questions remain about the purpose of mood monitoring interventions and how they might be improved to be most useful for people with depression. Qualitative methods offer good potential to elucidate some of these factors^12,16^.

The question remains how mood monitoring interventions could influence self-management procedures e.g. behaviour change and maintenance processes that may relate to multiple different recovery or lifestyle targets fundamental to living well with depression. Digital tools can be used to serve multiple purposes^17^ such as information seeking (e.g. I am not feeling quite right—is this low mood or is there another reason?), emotional support and reassurance (e.g. is my mood okay right now?) or for decision support (e.g. is now a good time for me to change job, get married or take on additional potential stressors?). These wide-ranging protocols often include additional features to ambulatory assessment^18,19^ and there is uncertainty about whether these components are valued by people with depression. Sharing of data is a common suggestion^19^ and there has been little qualitative evaluation of how intrusive users find these approaches, how concerned users are about their data being misused^20^, and therefore how closely protocols should be aligned with formal mental health services and to what degree they should be tools for self-management to be used in the personal sphere^17^.

This is the first systematic review to our knowledge that assesses user experience of mood monitoring and ambulatory assessment protocols. Specifically, we wish to focus on common themes that explore barriers and facilitators that may aid or hinder engagement with ambulatory assessment protocols. Where the tool is used as a mood monitoring intervention, we sought the intended purpose of the mood monitoring protocol. We explore certain overarching concepts that had particular salience to people with depression e.g. sharing with others^21–23^. We will explore these themes for both people with depression, for clinicians and for researchers. In this review we define users as anyone who might directly use the intervention or the information that arises from it. We define participants as those with depression and clinicians whose opinions are sought of their experiences with mood monitoring/ambulatory assessment in the context of the studies reviewed here.

We wished to explore how ambulatory assessment/mood monitoring affect people with depression (both positively and negatively from the perspectives of people with depression and clinicians). Considering the attrition and occasionally poor adherence to these interventions, we also aimed to explore under what circumstances use is maintained. We consider desired features of the protocol and practical ways of addressing barriers to uptake and continued use. We expand on these results to make a series of recommendations for the future development of these protocols to maximise acceptability and usability.

## Results

**Fig. 1:**
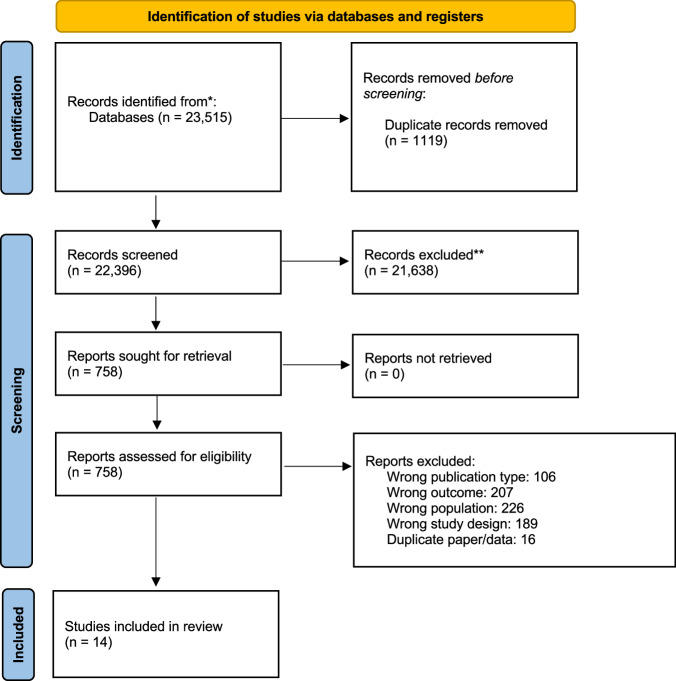
PRISMA flow diagram of included studies.

The search identified 23,515 papers. There were no studies reported in languages other than English that met the inclusion criteria. Following title and abstract screening, 21,638 were excluded, resulting in a total of 758 papers being reviewed in full. A total of 14 papers met the eligibility criteria and were included in the meta-synthesis. The other 21,638 were excluded as per Fig. 1—PRISMA flow diagram. The 14 included studies included 457 participants.

Supplementary Table 1 displays detailed characteristics of the studies and the ambulatory assessment protocols used. Supplementary Table 3 demonstrates the 7 different synthesised themes and provides exemplar first and second order constructs to illustrate these. Table 1 provides illustrative quotations for each sub-theme.

**Table 1:** Examples of illustrative first or second order constructs to demonstrate sub-themes and third order constructs

Third order construct: synthesis of main findings into an explanatory framework	Sub-theme	Illustrative first or second order construct – direct quotes from the relevant study
Negative psychological effects	Mood monitoring is confronting	I would be in a pretty decent mood, and I would complete the questionnaire accordingly, and then the results would indicate that I was actually in a much worse mood than I thought I was. This would make me doubt my ability to assess my inner state, which would put me in a worse mood.
	Compulsive monitoring	Perhaps a Fitbit could be almost quite negative, but, because I think you could maybe get a little bit obsessed with it.
	Decrease motivation	Whether the tool was providing new insights into mood-affected motivation to continue with regular completion; participants who reported being aware of influences and fluctuations in their mood reported most difficulties with motivation.
	Burden of mood monitoring	At the beginning, it was a bit difficult because I was working, then as I was on sick leave for two years, the truth is that I’ve been able to adapt quite well. And in the end, when I went back to work again, it was a bit difficult…
	Unwelcome reminder of mental illness	When you start to get worse, like it’s just disheartening. And then you have to go two twice through it, one for the therapist and one for the study. So that wasn’t great
Perceived effectiveness	No subsequent behaviour change	It’s informative. It doesn’t change my lifestyle
	No additional benefit	However, tracking mood did not generally provide new insights but only affirmed or validated what participants already knew. Most appreciated the affirmation: ‘It just affirms what you’ve been feeling in the day, so you can be like ‘hey I’m having a bad day, that’s alright ’, rather than worrying about it.’
	Participants consider it effective	You stop to think about it more. That nice things are really nice whereas you normally just take them for granted.
Difficulties in completing questionnaires	Difficult to track mood when unwell	It depends where I am mentally on that particular day. Um…sometimes, do you know, I won’t, I won’t, won’t be able to get out of bed to brush my teeth. And to be able, do you know, look onto your phone and fill out questionnaires, it’s nigh on impossible.
	Difficulty answering and interpreting the limited active ambulatory assessment measures	The answers are very closed, so you can’t really answer what you feel. You know? It’s very…it’s very up in the air.
	Active ambulatory assessment repetitive	It felt a bit too similar, um, every day. You know, ‘how did you sleep?’, ‘Yes, OK’, you know? It’s like the same sort of questions every day so it’s a little bit monotonous I think.
	Regular use is important	Participants acknowledged the importance of regular completion and recognised the potential for a distorted portrayal of mood with less regular completion.
Sharing with others	Direct access to a clinician through the app	Patients insisted on the need to be able to reach their physicians, or at least a mental healthcare professional each time they needed to, regardless of the time or day.
	Sharing with clinician improves understanding and communication	True Colours would be a way of keeping track of everything and also it would give me a visual representation to show medical professionals as opposed to just going well… I had a bad week 2 weeks ago. I can actually show them what happened as opposed to trying to remember it.
	Sharing with family/friends/people with depression creates community and provides support	This technology was also thought to provide an opportunity to create a community by sharing achievements and involving others in their goals, thus providing an additional source of support.
	Sharing depends on trust	In general, allowing trusted clinicians to view RMT data alongside medical records was acceptable, or even essential: ‘let’s say my whole history, my doctor already has it, if she has it more extensive, then all the better for me.’
	Clinician is not interested in the information	I thought it would be more relevant for my neurologist, but my neurologist wasn’t particularly interested when I told him about what I was doing in the study.
	Clinician could misinterpret data	I would want to make sure that my health record reflects actuality rather than something that can be interpreted by people incorrectly.
	Sharing feels intrusive	Attitudes toward the buddy system varied: some participants were concerned about the intrusive-ness of the system
	Don’t want to medicalise other relationships	Some participants were concerned about…the new dynamic it created with their friends.
	Don’t want to burden others	I think it’s a huge burden … I think they would’ve felt that they have to support me if they’ve seen all these thirties; they’d think I was suicidal and I wasn’t anywhere near it. I just think it wasn’t fair.
	Concerned about replacement of human contact by digital app	Any replacement of human interactions was almost universally considered detrimental to the therapeutic alliance.
Desired features	Other aspects in addition to mood monitoring	These brief interventions were designed by young people to be something that could be used regularly, rather than only when they were experiencing high distress. They could include all of the distraction interventions described above and also include features such as a photo album that contained meaningful photos, or photos that induced a positive emotion, supportive messages from friends and loved ones and messages that induced positive emotions, links to music playlists (eg, Spotify), and inspirational quotes (with the option of quotes generated by the user or app generated).
	Concerns around price and accessibility	Patients and physicians emphasised the need for a free app to ensure access for all patients with depression, regardless of their incomes.
	Crisis/wellness plan or procedure	Young people agreed that it was important that on every screen of the app there was a one-touch option to access emergency services for immediate crisis intervention, as well as a one-touch option to call or message (using a preprogramed default message) their support person, and a one-touch option to see their list of support contact people.
	Ease of use	A diagram or a histogram, well visible, easy to read. We need something more ludic, intuitive and easy.
	Graphical display of mood	When asked how they might wish to view their symptom data in future use, the majority felt that this was best displayed visually through in-app graphs.
	Reminder notifications to complete ambulatory assessment	I think like it’s good that there’s a reminder…and that I could choose when it was. Umm…because it’s like quite a convenient time for me just like in the evening to go on my phone, and it’s quite quick to do the questionnaires…and like you can choose when that comes which I think’s really good.
	Support required prior to/during use	To reduce the potential difficulties bound to the use of the app, physicians should plan a dedicated educational time with the patient to explain the functioning of the app. A user guide should also be included and delivered to the patient.
	Personalisation	The content and functioning of the app should be tailored to each patient and should adapt to the patient’s condition over time.
	Positive messages	I like recording what keeps me well, not what makes me ill. I’d much prefer contemplating to think more positively. To think, ‘oh these things work’ I like to keep focused on the positive side.
	Preference for the wearable device over smartphone app	Participants preferred mobile phone apps to wearable devices.
	Preference for digital methods	For aspects of therapy that cannot be fully automated, such as homework, participants still expressed a stronger disposition to engage with electronic methods.
	Full control over many aspects of the process/app data	In addition, patients wanted control over data collection and the option to engage with this technology or opt out rather than being a prerequisite for treatment.
	Effectiveness/scientific evaluation	Both patient groups discussed the issue of trust and whether technology was as effective as human-based treatments in the context of treatment. It was important for all groups that any recommendations based on digital tool readings be evidence-based or accredited by the NHS to ensure their reliability.
	Advantages of passive ambulatory assessment	Both patient groups expressed a strong dislike for repeated symptom measurement questionnaires, and automatic data collection was considered a pleasing alternative.
	Wearables comfortable	They’re quite bulky so they’re not very comfortable to wear at night.
	App feedback to not be patronising	I know, some people, it made them feel worse … it was a little bit patronising.
	Address technical issues	I think it is really important to have the practical support ‘cause you don’t want to be offline or not working for long than is necessary. Otherwise it goes against the purpose of the study really.
	Address data security concerns	Within the subtheme of security, participants were mainly concerned about sensitive information being leaked or picked up through digital sensors and going out to third parties and private corporations.
	Easily interpretable data/results	Many also expressed that this would need to be accompanied by ‘a human explanation for what those things mean.’
Purpose of mood monitoring	Provide accountability/goal setting	You could have it where like the therapist can see the log and they could see like ‘Oh, you did some activity at you know 5:30 when you finish work so I can see that you went for walk this day or maybe you didn’t go this day’. It stops people like me lying.
	Aid/monitor treatment	It felt like it’s just part of that - a process of evaluation, and the data was helpful when I needed to speak to my therapist about things.
	Improve insight	I’m more aware of it, the questions on the questionnaire, especially those that ask how I’m feeling right now raise my awareness.
	Memory aid e.g. medication adherence	Participants thought positively of RMTs as tools for memory aids in several ways, including helping manage practicalities such as taking medication and keeping track of what improves their mood.
	Monitor sleep/physical health	I was thinking probably when I don’t sleep well … that’s a sign. You can get these tracker things now and I was thinking getting one myself, that’s supposed to track your sleep. I thought maybe something as simple as that might actually be helpful.
	Improve motivation	I mean, like the steps I take have a direct effect on my health, both physical and mental, all my activity makes me more aware of it, more conscious of it and it has also been like a driving force for me to put my batteries in sport or stress management…a habit forever, so I do not want to do without it.
	Objectively judge mood	I think trends are really quite important for me in managing what is going on…I think one of the things I am thinking would be good to come out of this is an ability to see patterns over time and then maybe being able to use that as a predictor or, I need to do some intervention here so that I don’t end up there again if that makes sense.
	Reassurance	Knowing that there is somebody out there that’s monitoring me…which is nice.
	Relapse prevention	When participants in this consultation exercise were asked what they thought would be a successful measurement for the implementation of RMT, they endorsed the idea of detecting and predicting relapse.
	Improves self-management	I think trends are really quite important for me in managing what is going on…I think one of the things I am thinking would be good to come out of this is an ability to see patterns over time and then maybe being able to use that as a predictor or, I need to do some intervention here so that I don’t end up there again if that makes sense.
Clinician components/perceptions	Facilitates monitoring of treatment adherence	Clinicians spoke about how the mobile behavioural-sensing platform could help them confirm students’ compliance and adherence to the assigned homework.
	Improves clinical decision making	I’m big on physical activity when treating depression…the data can help me to see if they are complying with the 45 minutes four days a week, then I can say confidently that we have done a serious intervention that would like parallel what would happen with a medication like an SSRI.
	Improves efficiency of time with clinician	Seven participants suggested the feedback provided via True Colours (i.e. graphs) could improve the efficiency of time spent with healthcare providers.
	Enables positive reinforcement/feedback	I would like a portal for me to be able to go in and give them notes of encouragement. Like ‘Hey, John, saw your data today. You look great.’
	Decreases autonomy / managing expectations	Clinicians highlighted a potentially counterproductive effect of one feature of the app (the function allowing a preprogramed default message to be sent when a young person was distressed), in that it could create a learned helplessness with regard to help seeking.
	Difficulty in integrating into practice	Only physicians described the risk of time consumption associated with the use of the app as a strong limitation. They worried about not being able to deal with the app in addition to their other professional duties. They also expressed doubts about their ability to integrate these tools into their daily practice.
	Increased workload	Clinicians raised further concerns about whether RMT data would be used to evaluate risk and whether this would add to their workload.
	Limited clinical utility	At the same time, they warned that objective benchmarks for outcome definitions would vary drastically across patients, such that symptom improvement may manifest with opposite digital signatures across different conditions.
	Concerns about risk/liability issues	…it can be bad because if we missed something, or we can’t reach them, then we are still responsible for the information that we have. It can go both ways, but it’s just important to think about the liability piece.

These are all direct quotes from the study but not all of them are direct participant quotes—e.g. some are first order and some are second order constructs. We could not include direct participant quotes for all of these due to unavailability.

Fourteen studies used 7 different ambulatory assessment protocols. The ambulatory assessment protocols were: RADAR^13,14,24–26^, Moodscope^27^, ZELF-I^15^, True Colours^22^, iSee^23^, Co-HIVE^28^ and mobile texting programme^29^. The remaining six studies explored hypothetical use of an untrialled and unnamed ambulatory assessment protocol^21,23–25,30,31^. Seven studies assessed actual use of the protocol over periods from 3 to 12 months^13–15,22,27–29^. All studies included participants with depression, however, Bos et al.^30^, Simblett et al.^25^ and Simblett et al.^26^ used a mixed sample (Supplementary Table 1). All studies used adult samples apart from Hetrick et al.^21^ which used a sample of young people (age 16–25). All studies explored the user experience of ambulatory assessment/mood tracking protocols, or used implementation/co-production frameworks to try to improve the usability and acceptability of the protocol.

Supplementary Table 2 reports CASP risk of bias assessments. All of the included studies were judged to be of sufficient quality to contribute to the meta-synthesis. All papers reported their aims of the research clearly and were considered to be valuable in their contribution to the themes. Question 6 of the CASP referring to the relationship between the researcher and participants was not acknowledged in any of the studies.

The synthesis revealed seven overarching concepts: negative psychological effects, perceived effectiveness, difficulties in completing questionnaires, sharing with others, desired features purpose of mood monitoring, clinician barriers/facilitators. These can be summarised as barriers and facilitators to mood monitoring for both people with depression and for clinicians—and we focused on certain overarching concepts that seemed to have particular salience e.g. sharing with others. Supplementary Tables 4–9 report which studies endorsed which sub-theme.

### Negative psychological effects (Supplementary Table 4)

Eleven papers reported negative psychological effects data and we report five sub-themes here. The other three studies did not explore^26,28,29^. All 11 studies included participant reports of the protocol resulted in them confronting a worsening of their mood and/or anxiety. The protocol caused a sense of foreboding in some— this was usually when forewarning them of a possible dip in their mood and thus a relapse into depression. Users were concerned that this was a self-fulfilling prophecy and this foreboding would cause low mood. Some participants avoided self-reflection required by the questionnaires when feeling low, finding it patronising, too confronting or that it was worsening their sense of helplessness. For some this avoidance led to guilt. Some people with depression did not review their behavioural targets when it did not show any positive change because of a concern that this awareness would worsen their depression. Fundamentally, users wanted to record things that they felt kept them well rather than what makes them ill.

One participant reported that the protocol would frequently highlight their poor sleep quantity and quality on waking and that being reminded everyday of an ongoing problem was demotivating.

Some individuals reported worrying or even rumination about low mood after mood monitoring in two papers^22,24^ (de Angel et al.^24^: ‘Subthemes within self-awareness revolved around having a reflexive tool to identify triggers and boosters of well-being, assess treatment trajectory, positive or negative reinforcements, and the potential to worsen rumination and health anxiety’). Some reported obsessing over the data. For others, it was an additional task to carry out on a long to-do list. Users reported becoming negatively preoccupied with tracking their mood. This analysis of their depression, the causes of their depression and their feelings was seen as a distraction from ‘living [their] life’ and achieving something meaningful that would help them.

For some completing mood questionnaires daily and thus regularly reflecting on their mood was an unhelpful reminder of their depression, for example the assessments sometimes interrupted their positive mood.

### Perceived effectiveness (Supplementary Table 5)

Eight papers reported perceived effectiveness data and we report three sub-themes here. Users felt that mood monitoring was effective in improving their mood, giving them additional topics to discuss with their therapist and improving their experience of psychological therapy (e.g. ‘It felt like it’s just part of that - a process of evaluation, and the data was helpful when I needed to speak to my therapist about things’^13^). Other users reported that the mood monitoring boosted the treatments they were receiving alongside the monitoring. Mood monitoring use allowed people with depression to have an objective behavioural measure to contextualise their symptoms. This appeared to translate into improved insight and thus the incorporation of self-management strategies to prevent relapse of their depression. Some of these strategies related to lifestyle adjustments, motivation, accountability and reassurance e.g. conversations with trusted individuals about their mood, behaviour and feelings, considering how to improve their sleep, exercise, thinking carefully about an appropriate intervention to stop their mood worsening. These strategies appeared to be based on an iterative process with self-management techniques changing and adapting as the users gained insight into their condition through studying their mood data, self-reflection and discussions with their clinicians.

Some participants reported that the use of mood monitoring did not lead to any subsequent behaviour change. One user felt that they were not able to translate the information the app provided into self-management strategies. For some users the app provided no additional benefit and confirmed what they suspected or knew already. For some this was reassuring but for others this lack of new insight was not helpful and it was hard for them to maintain motivation for continued use.

### Difficulties in completing questionnaires (Supplementary Table 6)

Nine papers reported difficulties in completing questionnaires data and we report four sub-themes here. Many users found the questionnaires repetitive and disliked the negative phrasing of their symptoms. Users felt that there was limited value in answering additional questions per day e.g. one user reported that reporting their mood five times per day gave them little benefit when compared to three times per day. Questionnaires often came at inconvenient times when users could not answer them and it was difficult to integrate this into their routine. This barrier appeared specific to ambulatory assessment studies where participants did not have a choice about when they received the assessment^14,15^.

Some individuals reported that they struggled to complete the questionnaires when their mood was low due to a lack of motivation, cognitive dysfunction and anhedonia. Some suggested the possibility of postponing answering the questionnaires if they felt too low to complete them. Furthermore, the questionnaires were at times difficult to interpret. Participants were unsure how to respond and felt that some self-report items were very similar to each other or difficult to understand (e.g. ‘There was one question on there which…um…seemed a bit…could be misconstrued’^22^). Some felt that the answers were too closed, and they could not communicate their emotional experience. The data and information provided by the app was also challenging to interpret for some and required a human explanation of what these things meant. One person with depression felt that reviewing the data was a burden and a struggle to analyse large volumes of data to generate actionable insights. Some clinicians also agreed that people with depression would struggle to understand the information about their mood.

### Sharing with others (Supplementary Table 7)

Nine papers reported sharing with others data and we report 10 sub-themes here. Users had a wide range of opinions of sharing their data with clinicians, family and friends. This included whether they wanted to share or not, the amount of data shared and the timing of this.

For some users, the decision to share with their clinician was based on trust and a belief that their clinician having more information would improve the care of their depression. This was considered acceptable and even essential by some. Some users wished to choose what they shared with clinicians, while others felt that the clinician was in a better position to decide what was relevant.

Some people with depression reported attempting to share the information with their clinician, but their clinician was not perceived to be interested. Other users worried about their clinician interpreting the data without relevant context.

Some users expressed a strong desire to have direct access to a clinician throughout the ambulatory assessment, regardless of the time of day. This echoed clinician concerns about expectations around their availability.

People with depression felt that sharing their data with others helped them to have conversations with trusted others and their therapist. This improvement in communication was felt to be therapeutic. By sharing their achievements and involving other people in their goals, users felt they were creating an important supportive community. Users wished for control over what was shared and how/when. Some participants found that sharing their data with trusted individuals felt invasive at times and created a new dynamic with their friendships—potentially medicalising their close relationships.

Finally, people with depression were concerned about the replacement of human contact by a digital app—face-to-face conversations were felt to facilitate open and honest conversations about illness and aid recovery. Some felt that digital approaches lacked the accountability that a face-to-face conversation could provide.

### Desired features (Supplementary Table 8a, b)

Thirteen papers reported desired features data and we report 20 sub-themes here. In addition to mood monitoring users wished for additional features. These included: physical health tracking, psychoeducation, memory aids, pro-motivational features, more personalisation that is specifically tailored to an individual’s symptoms and treatment, lived experience testimonies, a separate section of the protocol dedicated to carers, a broader social network for people using the app including a forum and chat function, games, relaxation exercises, cognitive behavioural therapy exercises, distraction interventions, photo albums, supportive messages from friends, music playlists, inspirational quotes, emergency service contact, a one-touch method of alerting a close individual when in crisis, diary, phonebook, information about medication e.g. side effects and the incorporation of professional advice e.g. medication changes.

Users were concerned about potential access issues, including cost. Some suggested that the app be freely available to all while others felt that it should be prescribed or access recommended by a clinician. Concerns were raised that there were individuals who may be excluded from the tool—such as those who were less technologically competent or lacked the required smartphone/computer. Users expressed discomfort at potential marketing of the app and in-app advertising. Preferred alternative methods of funding were not explored.

The incorporation of a tailored crisis procedure was considered important. Users considered different methods for this: e.g. written advice provided by the app in times of crisis/symptom worsening or a call to their physician or family. Some users suggested a partnership with emergency services to provide a quick and reliable reaction to suicidal intent. Other users were aware of the constraints of services and did not expect clinicians to respond to distress messages out of hours, instead suggesting a one-touch method to call or message a trusted individual.

Participants emphasised ease of use and the solving of technical problems. Participants reported that passive data collection drained their battery to an unacceptable degree as well as errors and glitches in the app. Users reported using the protocol more when they felt the process was easier and more user-friendly. Participants expressed a desire for a graphical display of their mood to visualise changes over time. They were complimentary of notifications to remind them to complete self-report questionnaires. Some users preferred digitisation of the protocol as much as possible. Some participants preferred just smartphone-based protocols to those incorporating a wearable device.

Many individuals felt that they required some degree of guidance to use the app and to analyse the data that it generated. Some suggested a user guide, while others proposed a face-to-face session with their physician to explore how the protocol might be useful for them. Others wanted to only analyse the data with their doctor so that their physician could interpret it for them and provide advice/management suggestions.

Participants often had differences in preferences about how to interact with the app. There was a desired flexibility in when and how to receive feedback on their mood. Some users wished to receive less feedback, while others wanted more to improve the experience. People wished to omit personally irrelevant questions and add other items they felt had more relevance. Users were clear that these tools were not a one size fits all and that the lived experience and opinions of users should be accounted for during the development of the tools.

Users expressed a desire for positive reinforcement, for example, in the form of supportive messages. Individuals seemed to prefer to record what keeps them well rather than what keeps them ill. Positive messaging was seen as motivational and improved users’ self-esteem.

Some users wished to receive feedback on their data in real time in order to consider and potentially act on the information. Others were cautious that receiving data during periods of low mood would be detrimental and preferred to receive their data at the end of the protocol period.

People with depression felt that trust in the technology was important. It was important whether the protocols were as effective as face-to-face therapies in the treatment of their depression. For people with depression, accreditation by a trusted body such as the United Kingdom National Health Service and an appropriate evidence base for use was important. Furthermore, users wanted control over their data. They wanted to choose whether the protocol collected, recorded and shared certain pieces of data e.g. GPS data. Participants wanted the option about whether to and how to engage with the protocol as and when they wanted—they did not wish for use to be a prerequisite to accessing treatment. For some, this overlapped with concerns around data security.

Users report multiple technological problems with the software, hardware e.g. wearables. Some users reported battery issues with the device and software bugs/errors. Others reported issues with the protocol not being user-friendly—e.g. difficult to use, difficult to interpret, overly-complicated. The wearables were difficult to use, uncomfortable, too bulky. Some users reported their FitBit caused them bruises or they were allergic to the watch strap and so they wore it infrequently. Users were also concerned about data security and anonymity—in particular their private health data being leaked into the public domain or to third parties e.g. private companies. Some users did not feel privacy was a concern if their data was deidentified before secure storage.

Passive ambulatory assessment was felt to be one of the major improvements by replacing repetitive and negatively focused symptom questionnaires, which many users disliked. Passive ambulatory assessment was also positively viewed for replacing paper and pen techniques, gamifying data collection, convenient and automated use. Many users preferred wearables for passive data collection and after a short period of time, became accustomed to continuous wear and stopped noticing it. The separate device seemed to give some participants a sense of awareness and control over when the data collection took place. Users emphasised that regular reporting of their mood or compliance with the passive data collection was importation to provide an accurate estimate of their mood.

### Purpose of mood monitoring (Supplementary Table 9)

All 14 papers reported purpose of mood monitoring data and we report 10 sub-themes here. Users felt that the mood monitoring provided beneficial accountability to their clinician or therapist. One user expressed that when their clinician could also see this data, it stopped them being untruthful about whether they had done their assigned CBT homework. The mood monitoring protocol also provided objective measurable targets and goals for some users.

Users felt that the mood monitoring protocol improved compliance and adherence with treatment and medication. Sharing concrete information about their mood and symptoms with their clinician was felt to improve their treatment. The mood monitoring was felt to be helpful in determining the next treatment decision, both for medication and also in terms of lifestyle changes and psychological therapy.

People with depression felt their insight had been improved as a result of the mood monitoring. This appeared to be underpinned by having an objective behavioural measure to contextualise their symptoms (e.g. the app providing feedback on their mood or the participant realising this themselves). This insight appeared to translate into the incorporating of self-management strategies (e.g. conversations with trusted individuals about what had influenced their actions and feelings) and to prevent relapse of depressive symptoms. Users were more aware of their daily habits, their physical and mental health. This improved their motivation to continue to monitor their mood. Users felt that they were more conscious of their mood and of positive experiences in their lives, and that the data forced them to analyse potential triggers for shifts in their low mood. Users also wished to monitor their sleep objectively and reported that changes in sleep would cause a shift in their management of their mood. This multitude of data was highly motivational for some—particularly if they were managing to achieve their goals/targets already. This retrospective judgement was useful in participants assessing their mood over the long term and in sharing this with their clinicians. Many users found this monitoring to be very reassuring.

Participants used mood monitoring as a memory aid to remind them to take their medication and remember what improves their mood.

### Clinician barriers and facilitators (Supplementary Table 10)

Eleven papers reported clinician barriers and facilitators data and we report 10 sub-themes here. Some clinicians were able to use the ambulatory assessment to monitor and confirm adherence to medication/CBT homework. This helped assess whether the intended intervention has been delivered or not (e.g. ‘I’m big on physical activity when treating depression…the data can help me to see if they are complying with the 45 min 4 days a week, then I can say confidently that we have done a serious intervention’^23^).

Some clinicians felt that this accountability improved the adherence of people with depression. The data from the ambulatory assessment was used by clinicians to set more appropriate CBT homework based on what they felt the person with depression was able to do. This was seen as clinically important in setting achievable homework that did not decrease motivation if the participant failed to complete it. Clinicians also felt that the mood monitoring improved the time efficiency of the consultation—the mood data was already available, and they did not have to spend time taking a history of recent mood changes.

Clinicians were enthusiastic about positive reinforcement messages to people with depression. Some clinicians felt that this validation improved motivation and self-esteem and that a method of clinicians or the ambulatory assessment providing positive feedback was important to the treatment. When clinicians were concerned about progress or a potential deterioration in mood they recommended a supportive framing of reminders. Rather than showing them behavioural targets that might be difficult to achieve and thus demoralising they recommended gentle reminders used to encourage self-management, reflection and reassurance.

## Discussion

This systematic review assessed the participant, user and clinician experience of mood monitoring and ambulatory assessment protocols. We investigated facilitators and barriers, including negative psychological effects, to mood monitoring for both people with depression and for clinicians. We considered pragmatic ways of addressing these barriers to use by considering requested and desired features—these recommendations are summarised in Table 2. While many users found the ambulatory assessment therapeutic and positive, other users perceived the protocols negatively. There appeared to be significant room to improve on existing protocols.

**Table 2:** Recommendations and practical implications for future ambulatory assessment protocols

Theme	Recommendation	Practical implications
Negative psychological effects	- Ask about negative psychological effects of ambulatory assessment.	- If they are found then determine if they are temporary and mildly distressing or longer lasting and more severe.
User choice and personalisation	- Consider allowing users to opt into the different methods of data collection e.g. via active or passive ambulatory assessment. - Have the option to select from different methods of personalised feedback that suit them, the data they choose to track their self-management strategies.	- Giving such a choice may be impractical, introducing potential measurement bias. - Increasing flexibility and personalisation, however, may be expensive, technologically demanding and potentially less reliable in certain respects than a more uniform technology.
Data security and artificial intelligence	- Sharing of data be opt in rather than occurring automatically. - Data should not be shared with external organisations/companies without prior consent.	- Increasing personalisation is likely to involve the use of machine learning models and artificial intelligence. Some people with depression and clinicians might be suspicious of these approaches—particularly concerning a propensity to serious errors^63,64^. - Using artificial intelligence would need rigorous testing versus more conventional gold standard approaches.
Adaptability and personal relevance	- Adaptability of ambulatory assessment content to people with depression, to use alongside their existing treatment.	- The emphasis on personalisation and flexibility reported here resonates with similar research assessing the user experience of other digital health interventions specifically for individuals with somatisation disorders^65^ and anxiety^57^, as well as the public and health professionals more broadly^66^. - Incorporating personalisation and flexibility may create a sense of ‘self’ in the digital health intervention—a sense that this personalisation is relatable and applies to their own situation and life^66^—potentially improving engagement^57^ and overall perception^65,67^
Repeated measurement and user burden	- Consider the form of repeated measurement used to avoid repetition and negative phrasing/decreased motivation. - Consider alternative measures of mood to the existing validated self-report questionnaires trialled in the protocols reviewed here. - Incorporate more positive feedback and reassurance into personalised feedback strategies based on mood data.	- Other measures of mood include visual analogue scales or more usable and acceptable measures that aim to decrease potential participant burden. - Measuring depression using strengths may prove difficult, but it is possible that some alternative measures of mood may not cause individuals to feel that they are dwelling on their low mood.
Customisation of notifications and user burden	- Customisation of notifications is important to assist with user burden and to enable users to keep a routine alongside this.	- Incorporate notifications to complete the ambulatory assessment but include flexibility about when the notification occurs and when the ambulatory assessment can be completed.
Trust and scientific validity	- Trust in the scientific validity of the protocol is fundamental and future ambulatory assessment protocols should advertise this e.g. both effectiveness and in comparison to alternative psychotherapies. This may include formal accreditation from trusted bodies to support this. - Evaluate and consider the performance of assessment protocols in their development, as it appears to be the core functionality of many protocols.	- The performance of the ambulatory assessment protocol is important^45^ because many of the purposes of the ambulatory assessment/mood monitoring (e.g. lifestyle interventions/self-management) depend on the good performance of the protocol and accuracy of the data. - The data should be of high quality and accuracy and this could lead to improvements in effectiveness (e.g. insight, understanding and awareness of mood). This might lead to positive behaviour change and improvement in mood provided that the approach seems supportive and not overwhelming. - Other research in both people with depression and clinicians treating depression^68–70^ have indicated the requirement for validity, reliability and precision in the measure of mood.
Diversity and conflicting user needs	- Consider the multiple needs of a diverse group of individuals.	- Incorporating this diversity of views (e.g. contradictory opinions) will be a major challenge for future protocol development, but may involve customisation and personalisation. - Additional restrictions to address data security concerns would likely come at a cost of causing the active logging of data to be slightly more cumbersome. This runs in contrast to other users for whom ease of inputting their data was fundamental.

The vast majority of studies included more than one participant reporting finding the ambulatory assessment confronting, whether through realisation that their mood was lower than they realised, a sense of foreboding, ruminating about their low mood or obsessively checking their mood. There was evidence of temporary but not persistent distress—it was unclear if these negative feelings were only momentary or if this produced a longer-term change of mood in some individuals, indicating the challenges of self-measuring/interpreting the dynamic nature of mood states^32^. People with low motivation might especially struggle with such distress. However, mechanisms such as rumination and persistent worry about low mood may potentially lead to more persistent low mood through mood monitoring^33^. Therefore, future studies should directly ask about distress from mood monitoring and its persistence, and consider steps to reduce severe or persisting distress following mood monitoring. This review builds on existing research by summarising the qualitative reports of negative psychological effects in the absence of definitive quantitative evidence^34^. Quantitative ambulatory assessment/mood tracking studies, however, have so far not demonstrated a long-term worsening of depressive mood in participants^34–37^, suggesting that the negative feelings from mood monitoring are likely to be temporary. However, we note that most of these quantitative studies do not routinely report negative psychological effects^34^; as a result, we do not know if they have not asked about negative psychological effects or reflects that they are uncommon. Many users reported a worsening of their mood and anxiety due to mood tracking, while others reported benefits in terms of improved insight, behaviour change and subsequent relapse prevention. These negative psychological effects will have to be considered carefully when choosing who may or may not benefit from the intervention^38^. For example, those who already reported having good insight into the fluctuations of their mood reported most difficulties in motivation, contrary to many studies where insight often improves motivation^39^.

Less burdensome digital methods, such as the incorporation of passive ambulatory assessment, may solve some of the barriers to use and negative psychological effects (such as the repetitive nature of the assessments), but others are likely to be more central to the concept of mood monitoring in depression—such as the possibility that ambulatory assessment may worsen some users’ mood and anxiety^38^. People with depression may require additional support to avoid a worsening of their mood during ambulatory assessment e.g. additional techniques to tackle negative automatic thoughts^40,41^. It is possible that when mood monitoring is used as part of a therapeutic process, such as psychological therapy, or if it enables new insight and reflection, then there can be benefits to the person’s mood either through self-management or collaboratively with a clinician or trusted individual. Since depression is associated with low self-worth, self-activation and self-actualisation^42^, it is possible to see how mood monitoring allied to a series of behavioural goals and positive encouraging statements might have the therapeutic potential to improve depression through a sense of mastery and achievement^43^. Mood monitoring on its own or without such aims, however, might have either no effect or even make depression worse by reminding the person of their lack of achievement and how awful their subjective experience of depression is. Participants reported here that mood monitoring/ambulatory assessment gave them additional topics to discuss with their therapist. It may be that the mood monitoring was beneficial when allied to a purpose when having psychological therapy (e.g. contextual factors—fitting the monitoring in with other information - what else was going on in their lives?), but less helpful when it is done without a clear therapeutic opportunity. This was potentially reinforced by many participants placing a strong emphasis on other features in addition to mood monitoring—e.g the allying of contextual information to prompt learning and insight.

The self-management techniques explored here were highly varied, so it is impractical to include them in the mood monitoring protocol itself. However, in some instances, the protocol could provide the data to allow the user to utilise it to deploy their own self-management strategies in a highly personalised way. Thus, the protocol might allow a high degree of flexibility and personalisation, and this could be achieved in a variety of different ways. It is possible that the technology might be less personalised, but the protocol in relation to how the information is used might be personalised. If the technology is more personalised, then that might include some of the functions being switched off for some people. This flexibility and personalisation, however, will also not be possible in all settings, as research will require the same protocol to be delivered to each participant. Additionally, some studies will be specifically investigating psychological treatment approaches to managing mood. There was also no data on how researchers experience the use of ambulatory assessment/mood monitoring for academic purposes.

The results present important ethical considerations in relation to the sharing of sensitive information and their role within clinical services and provide some insight into how to practically manage these. These ethical considerations revolved around data security and data sharing—e.g. with clinicians or with external organisations/companies. Many users felt that sharing the data with their clinician was essential to the purpose of the ambulatory assessment, while others wished to appraise their data prior to sharing it. There was a divergence of views about whether these intervention protocols should be incorporated into, or sit outside conventional mental health services. There was some suspicion about the possibility of misinterpretation of mood data by clinicians, but in general, scepticism about sharing this data with mental health services was minimal.

Users were clear that they did not wish for the protocol to replace face-to-face contact with a clinician, but to supplement it. This potentially raises issues about the business case for such protocols as the digitisation of healthcare strives to make efficiencies by decreasing face-to-face contact with clinicians. Previous research has suggested that once a good interactive relationship is established between the participant and the clinical research team, then further digital interactions were seen in that good therapeutic relationship, so less face-to-face contact could be achieved^44^. It is possible that digital methods might increase efficiency but only once a positive therapeutic relationship has been established. Users and clinicians both expressed concerns about interpreting the data and requested onboarding. Support may be necessary for people with depression to maximally use the protocol—particularly if it requires a degree of insight and reflexivity to apply the data to the users’ own self-management strategies. Personalised procedures appeared to improve motivation and perceived value and this may improve attrition and adherence to the protocols, which is often of concern^45^.

This is the first systematic review that has assessed the user, participant and clinician experience of mood monitoring and ambulatory assessment protocols (to our knowledge). Our results were checked for coherence by someone with lived experience of depression—acknowledging that PPI input is vital for the successful implementation of this technology^46^. We followed Cochrane review standards to conduct a methodologically robust review.

This review combined multiple heterogenous studies, and this is a common limitation of many systematic reviews. We assess a wide range of user experiences across a wide range of protocols—some of which are more restrictive or unfocussed that others. Some user experiences may only apply to a specific type of protocol and not to others. Some of the sub-themes in our results relate to specific studies and protocols that may be problematic in some important ways. For example, we included studies evaluating hypothetical use of ambulatory assessment protocols—6/11 of the studies evaluated here explored hypothetical use. It remains possible that these views may not apply to pragmatic use and real-life testing. Furthermore, some of these sub-themes may be power-related and some studies reported small numbers of participants. There were insufficient studies when considering the diversity of possible contextual factors that might influence participant and clinician experience of mood monitoring in depression, when considering a broad range of factors. These include digital and non-digital wearable versus non-wearable, active and passive, EMA versus non-EMA, in and without therapeutic intervention, human, avatar or digital supported interventions, lack of lower and middle income countries, young and old, severity of depression.

Despite this review being a comparison of a wide range of ambulatory assessment protocols, nonetheless these all included frequent mood assessment at the core of the procedure. In spite of the wide range of protocol differences many themes and sub-themes were shared across numerous studies, which support the relevancy of our findings. Other sub-themes, particularly in the clinician barriers/facilitators and sharing mood monitoring data, were only found in one or two studies. All themes were discussed in the majority of papers included here. We did not include any non-English language papers, although they were not excluded from the search. As some of the main sources of technology production are currently in China, India, South Korea and Japan, it is possible that we may have missed some relevant studies.

By reviewing the user experience of mood monitoring and ambulatory assessment protocols we demonstrate the great potential to improve existing protocols to maximise acceptability, engagement, adherence, user experience and usability. These range from technological solutions, performance issues, liaison with external organisations to relationship with mental health services and the incorporation of additional therapeutic elements to manage negative psychological effects. Fundamentally, users desired an intuitive and easy-to-use passive data protocol that build on their existing strengths with a high emphasis on personalisation and customisability. This allows the user to self-manage their depression in their own way or share their data with a clinician to improve their existing clinical care. Users wished to retain control over their data and the personalisation included: data collection, data sharing, feedback provided and interaction methods with the protocol e.g. notifications/wearables. We recommend that personalisation be a core feature of any future protocol development to maximise successful implementation and uptake of future protocols.

There were negative psychological effects from ambulatory assessment reported by multiple participants from the vast majority of studies so future protocols should monitor for negative psychological effects of mood monitoring to determine if they are temporary and mild or more long-lasting and severe. They might also consider testing the incorporation of additional therapeutic elements to manage such negative psychological effects.

## Methods

We adopted a methodology based on the Cochrane Handbook for Systematic Reviews of Interventions and completed a Preferred Reporting Items for Systematic Reviews and Meta-Analysis (PRISMA) checklist. The study was pre-registered with the International Prospective Register of Systematic Reviews (PROSPERO: CRD42023396473^47^).

### Inclusion criteria

We included studies that met the following criteria: qualitative studies exploring user perspective of mood monitoring/ambulatory assessment/EMA in people with depression. We defined depression as a current or previous clinical diagnosis, a self-report current or previous clinical diagnosis, meeting research criteria for a depressive disorder. Self-reported depressive symptoms and depression in the context of bipolar disorder were excluded as the user experience was theorised to be distinct. We included actual use and hypothetical use of ambulatory assessment/mood monitoring for depressive symptoms. The studies could be published in any language (although we did not include any non-English language papers) and could be digital or non-digital, although only digital studies were included. We searched the grey literature (e.g. conference abstracts, dissertations, policy literature, reports via ProQuest and Google Scholar—full details below) for unpublished studies which were eligible for inclusion, acknowledging that relevant computer science studies may be published here. There were no date criteria and we included studies including participants of any age.

### Search strategy and selection criteria

The complete search strategy in listed in the appendix. We searched Ovid, MEDLINE, EMBASE, PsychINFO, SCOPUS, IEE Xplore, Proquest SciTech Collection, Proquest dissertations and theses global, Google Scholar using the search terms. The search was conducted on 28/10/24. All abstracts were appraised by two independent screeners (L.A.W., G.M., G.S., D.P., R.P. and M.M.), and any disagreements were discussed, and a consensus arrived upon, with adjudication by a third independent screener if required. We acquired the full text of any potentially relevant papers and if we were unable to source the full text of the study, we contacted the corresponding author to request the paper. To determine if potentially relevant studies met the inclusion criteria the full text was reviewed separately by two authors, again with discussion and consensus with a third reviewer if necessary. All papers for inclusion were reference checked along with relevant systematic reviews^34,45,48–55^. Key authors were also emailed to see if the inclusion of any ongoing unpublished studies could be included.

### Data extraction

Two independent reviewers extracted data from studies meeting the inclusion criteria using identical data extraction forms. The form was piloted on 3 papers initially and adjustments were made. Irregularities in the data extraction were discussed and any discrepancy resolved with discussion.

### Assessment of study bias

We assessed risk of bias in each study using the Critical Appraisal Skills Programme Checklist^56^. Risk of bias was assessed by two independent reviewers (L.A.W. and S.P.). We did not exclude studies of low quality as we wished to include papers containing in depth data collection and analysis, acknowledging that this might provide valuable information regarding the user experience. We included papers that collected data through focus groups, individual interviews, semi-structured interviews and exit questionnaires. If studies collected data through open response text, we included them if there was richness in the data provided.

### Synthesis of results

First order constructs were defined as direct participant quotes reported in the papers^57^. Second order constructs were defined as the authors’ interpretations of participants’ quotes expressed as themes, extracted from both the results and discussion sections of papers in order to capture all of the constructs^57^. Third order constructs refer to the current authors’ further analysis of the first and second order constructs^57,58^.

Papers were read and re-read by L.A.W. and S.P. First and second order constructs were extracted and managed using Microsoft Excel. Any disagreements were discussed and consensus agreed on. Constructs were reviewed to assess how the themes juxtaposed and compared across papers. Reviewers independently reviewed the second order constructs, compiling third order constructs that summarised and encapsulated the various themes across the studies using NVIVO 12^59^. Discussion between researchers then refined these constructs until a consensual understanding was reached.

Noblit & Hare’s guidelines for meta-ethnography were used to conduct the analysis^60^. Noblit and Hare proposed three ways to synthesise data. 1. Reciprocal translation (where the findings of one study are understood in terms of findings expressed in other studies in the synthesis^61^) if the data is directly comparable. 2. If the data is in opposition, using refutational translation (which explains and explores inconsistencies, exceptions, incongruities in the data between studies^62^). 3. An integrating scheme can be produced which makes sense of the parts—a ‘line of argument’ which uses both similarities and differences across the studies. This assessment of the included studies showed consistent themes in additional to apparent contradictions in the user experience of ambulatory assessment/mood monitoring protocols. Due to this, we used the line of argument approach to make sense of apparent contradictions in the data to then integrate the emergent concepts into a framework of user experience.

Our results were checked for coherence by someone with lived experience of depression and over a decade of experience in patient and public involvement with experience of using and codesigning mood monitoring tools. While the research team was diverse some members were psychiatrists and therefore might have carried certain assumptions or biases about the delivery of ambulatory assessment/mood monitoring interventions in people with depression – this positionality may have impacted interpretation of the results via meta-synthesis.

## Supplementary information

Supplementary information

## Data Availability

As this is a systematic review of published work the data is freely available.
