# Supplementary material for: The user experience of ambulatory assessment and mood monitoring in depression: a systematic review & meta-synthesis
Source: NPJ Digit Med. 2025 Dec 2;8:737. doi: 10.1038/s41746-025-02118-8 (PMC12672782; doi:10.1038/s41746-025-02118-8)
Supplement: Supplementary file 1 — Supplementary information [file 41746_2025_2118_MOESM1_ESM.pdf]

Supplementary Information 1: supplementary tables 1 & 2

Study	Country	Sample	n	Mean age in years (SD)	% female	Intervention	Setting	Active vs passive monitoring	Ambulatory assessment/ mood tracking procedure	Ambulatory assessment duration	Generalisation vs personalisation	Person with depression led vs clinician led protocol	Time point of data collection	Data collection method	Data analysis method
Bos et al 2019	Netherlands	Depression: 45%, Bipolar Disorder: 32%, Anxiety disorder: 18%, Psychosis: 14%, Eating disorder: 5%, Autusm spectrum disorder: 5%, Unknown: 5%	Patients: 22, Clinicians: 21	20-35 years, n = 6, 36-50 years, n = 7, 51-65, n = 7, 66 or older, n = 2	64	Open enquiry into mood monitoring/tracking applications	Mixed sample – participants recruited via secondary care outpatients and mental health institutions.	Active	Hypothetical app where individuals can record their moods, experiences, behaviors, contexts, and thoughts multiple times per day on their smart phones.	Hypothetical use	Personalisation	Person with depression and clinician led	Hypothetical use	Semi-structured interview	Thematic analysis
de Angel et al 2022	UK	Adults with depression: 16, Clinicians: 6	22	44.6 (13.3)	82	RADAR-base system	Primary care - IAPT/NHS Talking therapies	Passive	Hypothetical discussion around RADAR-base passive monitoring	Hypothetical discussion around RADAR-base passive monitoring	Personalisation	Person with depression and clinician led	Cross-sectional	Focus groups	Inductive thematic analysis
de Angel et al 2023	UK	Adults with depression	66	34.6 (11.1)	61	RADAR-base system	Primary care - IAPT/NHS Talking therapies	Active & passive	RADAR-base system PHQ-8, RSES, speech task – weekly. Heartrate, step-count, GPS location, acceleration, light levels, phone interaction, nearby Bluetooth device detection, battery level, weather, sleep, app usage metrics – continuous	7 months	Personalisation	Person with depression and clinician led	Study end	Semi-structured interviews	Deductive thematic analysis

									via Fitbit/RADAR-base app.						
Drake et al 2012	UK	Adults with depression	16	Completers: 38.9 (12.6), Dropouts: 32.8 (15.3)	75	Moodscope	Primary care	Active	PHQ-9, GAD-7 - weekly. Moodscape self-rated mood - selecting which of each 20 interactive mood-adjective playing cards describes current mood.	3 months	Generalisation	Clinician led	Study end	Questionnaire, focus groups	Descriptive statistics, thematic analysis
Folkersma et al 2021	UK	Adults with depression	20	18-30: 50%, 31-45: 20%, 46-65: 30%	35	ZELF-I	Secondary care	Active	ZELF-I: 5 brief questionnaires per day for 28 days	1 month	Generalisation	Clinician led	Study end	Semi-structured interviews	Thematic content analysis
Hetrick et al 2018	Australia	Young people with depression: 11, Clinicians: 16	27	21.4	73	Customisable mood monitoring	Mixed sample – participants recruited via secondary care/advertising	Active	User centred development of mood monitoring app	Minimal use - Hypothetical discussion around development	Personalisation	Person with depression and clinician led	Cross-sectional	Codesign workshops	General inductive approach
Incecik et al 2020	UK	Adults with treatment resistant depression	21	41.2 (15.2)	38	True Colours	Mixed sample – participants recruited via secondary care/advertising	Active	True Colours - ASRM/QIDS-SR16 delivered via weekly SMS/email	1 year	Generalisation	Clinician led	Study end	Semi-structured interviews	Thematic analysis

Meng et al 2018	USA	Adults with depression: 12, Clinicians: 9	21	Adults with depression: 21 (1.22), Clinicians: 42 (5.83)	Adults with depression: 58, Clinicians: 89	iSee - conceptual prototype	Primary care	Passive	iSee - conceptual prototype using passive ambulatory assessment	Minimal use - Hypothetical discussion around development	Personalisatio n	Clinician led	Cross-sectional	Semi-structured interviews	Inductive thematic analysis
Patoz et al 2021	France	Adults with depression: 24, Clinicians: 26	50	Adults with depression: 51.5 (15.5), Clinicians: 45.5 (12.2)	Adults with depression: 54.1, Clinicians: 50.0	Hypothetical depression app	Secondary care	Active	Hypothetical depression app	Minimal use - Hypothetical discussion around development	Generalisation	Person with depression led	Cross-sectional	Semi-structured interviews	Content analysis
Simblett et al 2020	UK	Adults with depression: 8, Epilepsy: 7, Multiple sclerosis: 9	24	Adults with depression: 51.9 (9.4), Epilepsy: 44.4 (15.8), Multiple sclerosis: 43.4 (9.5)	Adults with depression: 63, Epilepsy: 71, Multiple sclerosis: 67	RADAR-base system	Mixed sample – participants recruited via advertising	Active & passive	RADAR-base system PHQ-8, RSES, speech task – weekly. Heartrate, step-count, GPS location, acceleration, light levels, phone interaction, nearby Bluetooth device detection, battery level, weather, sleep, app usage metrics – continuous via Fitbit/RADAR-base app.	Minimal use - Hypothetical discussion around development	Personalisatio n	Person with depression and clinician led	Cross-sectional	Focus groups	Systematic thematic analysis

Simblett et al 2024	UK	Adults with depression: 17, Epilepsy: 11, Multiple Sclerosis: 17 (Only data on depression included here)	17	55 (13)	83	RADAR-base system	Mixed sample – participants recruited via advertising	Active & passive	RADAR-base system PHQ-8, RSES, speech task – weekly. Heartrate, step-count, GPS location, acceleration, light levels, phone interaction, nearby Bluetooth device detection, battery level, weather, sleep, app usage metrics – continuous via Fitbit/RADAR-base app.	Minimal use - Hypothetical discussion around development	Personalisation	Person with depression and clinician led	Cross-sectional	Focus groups	Thematic analysis
Thomson et al 2024	Austrailia	Adults with depression	10	26 (Q1 - Q3: 20 - 50)	70	Co-HIVE	Secondary care	Active & passive	Co-HIVE system: smartphone application (Mentegram - administering PHQ-9, HDRS, BDI-II, DASS-21, C-SSRS) and smartwatch device for remote health monitoring (Fitbit Charge 5 - heart rate, stepcount, sleep), telehealth appointments (HealthDirect) for virtual check-ins/health coaching	10-12 weeks	Generalisation	Clinician led	Study end	Semi-structured interviews	Thematic analysis - framework method

Van Tiem et al 2021	USA	Adults with depression	21	48 (range: 28-72)	19	Daily test messaging intervention	Veterans Affairs	Active	Daily test messaging intervention including mood monitoring - daily text message exchanges with an automated system and weekly secure messages with visual representations of the participants messages. Linked to electronic health record and can be viewed by clinician.	12w	Generalisation	Clinician led	Cross-sectional	Semi-structured interviews	Inductive and deductive coding
White et al 2023	UK, Spain, Netherlands	Adults with depression	99	Time point - 3 months: 44.6 (12.1), 12 months: 49.4 (13.5), 24 months: 51.9 (15.0)	Time point - 3 months: 75, 12 months: 76, 24 months: 69	RADAR-base system	Mixed sample – participants recruited via secondary care/advertising	Active & passive	RADAR-base system PHQ-8, RSES, speech task – weekly. Heartrate, step-count, GPS location, acceleration, light levels, phone interaction, nearby Bluetooth device detection, battery level, weather, sleep, app usage metrics – continuous via Fitbit/RADAR-base app.	2 years	Personalisation	Person with depression and clinician led	3, 12, 24 months	Semi-structured interviews	Thematic analysis

Supplementary Table 1: included studies and their ambulatory assessment protocols

Study	Assessment of Study Quality									
	Was there a clear statement of the aims of the research?	Is a qualitative methodology appropriate?	Was the research design appropriate to address the aims of the research?	Was the recruitment strategy appropriate to the aims of the research?	Was the data collected in a way that addressed the research issue?	Has the relationship between researcher and participants been adequately considered?	Have ethical issues been taken into consideration?	Was the data analysis sufficiently rigorous?	Is there a clear statement of findings?	How valuable is the research?
Bos et al 2019	1	1	1	1	1	0	1	1	1	1
de Angel et al 2022	1	1	1	1	1	0	1	1	1	1
de Angel et al 2023	1	1	1	1	1	0	1	1	1	1
Drake et al 2012	1	1	1	1	1	0	1	1	1	1
Folkersma et al 2021	1	1	1	1	1	0	1	1	1	1
Hetrick et al 2018	1	1	1	1	1	0	1	1	1	1
Incecik et al 2020	1	1	1	1	1	0	1	1	1	1
Meng et al 2018	1	1	1	1	1	0	1	1	1	1
Patoz et al 2021	1	1	1	1	1	0	1	1	1	1
Simblett et al 2020	1	1	1	1	1	0	1	1	1	1
Simblett et al 2024	1	1	1	1	1	0	1	1	1	1
Thomson et al 2024	1	1	1	1	1	0	1	1	1	1
Van Tiem et al 2021	1	1	1	1	1	0	1	0	1	1
White et al 2023	1	1	1	1	1	0	1	1	1	1

Supplementary Table 2: Risk of bias assessments for included studies

Supplementary Information 2: Results in full

Supplementary Table 3: Examples of first- and second-order constructs and synthesised themes			
**Third order construct: synthesis of main findings into an explanatory framework**	**Sub-theme**	**Second order constructs**	**First order construct**
**Negative psychological effects** - some participants report a worsening of mood and anxiety when mood monitoring and consideration of individual coping style may predict who is more likely to experience this worsening of mood.	Mood monitoring is confronting	The interplay between mental health and engagement was found to be bidirectional. For example, when participants were unwell, some reported avoiding the self-reflection required by the questionnaires, whereas others experienced this only when feeling well. On the one hand, some reported an improvement through the encouragement of health-promoting behaviors, whereas others experienced guilt or anxiety from obsessing over data, especially if no improvement was apparent. (13)	"When you start to get worse, like it's just disheartening. And then you have to go two - twice through it, one for the therapist and one for the study. So that wasn't great." (13)
**Perceived effectiveness** – many participants considered the mood monitoring effective in improving their mood	Participants consider it effective	Participants, for instance, reported becoming more conscious of internally experienced changes (e.g., shifts towards more positive mood states) as well as of good things happening in the outside world (e.g., a pleasant encounter). (15)	"You stop to think about it more. That nice things are really nice whereas you normally just take them for granted" (15)
**Difficulties in completing questionnaires** - some participants struggled to complete active ambulatory assessment measures as a result of their low mood.	Difficult to track mood when unwell	Even setting aside the issue of time, over half of participants indicated that because of a lack of interest, not prioritising or viewing True Colours as useful and/or an inertia (related to their illness), it was difficult to engage consistently. (22)	"It depends where I am mentally on that particular day. Um. . .sometimes, do you know, I won't, I won't, won't be able to get out of bed to brush my teeth. And to be able, do you know, look onto your phone and fill out questionnaires, it's nigh on impossible" (22)
**Sharing with others** – sharing mood monitoring data with a clinician can aid treatment via therapeutic and reflective conversations.	Sharing with clinician improves understanding and communication	Interplay with treatment - improved perceived effectiveness: a) increases accountability; b) helps with homework; c) helped conversation with therapist. (13)	"It felt like it's just part of that - a process of evaluation, and the data was helpful when I needed to speak to my therapist about things" (13)
**Desired features** - participants were positive of reminder notifications but expressed a desire for control over the timing/quantity of these.	Reminder notifications to complete ambulatory assessment	Positive features relating to the interface were identified. Just over half of participants described the ease of logging on and completing questionnaire(s), and five participants commented on	"I think like it's good that there's a reminder. . .and that I could choose when it was. Umm...because it's like quite a convenient time for me just like in the evening to go on my phone, and it's quite quick to do

		the usefulness of personalised weekly prompts. (22)	the questionnaires...and like you can choose when that comes which I think's really good" (22)
**Purpose of app** - participants often used the data from the app to gain insight into their own patterns of mood and used this information to self-manage their illness in ways that were helpful to them.	Self-management	It was generally accepted that having access to data of this nature would be useful for both self-management and integration into clinical care. (14)	"I think trends are really quite important for me in managing what is going on...I think one of the things I am thinking would be good to come out of this is an ability to see patterns over time and then maybe being able to use that as a predictor or, I need to do some intervention here so that I don't end up there again if that makes sense. "(14)
**Clinician barriers and clinician facilitators** - clinicians expressed concerns about the expectations of them to respond to patients on the app and this associated increased workload.	Barrier: increased workload	Only physicians described the risk of time consumption associated with the use of the app as a strong limitation. They worried about not being able to deal with the app in addition to their other professional duties. They also expressed doubts about their ability to integrate these tools into their daily practice. (50)	"Let's take email: I read it once every two days. I cannot check it more often. I come back home really late, 11.30 pm sometimes. Checking mail and SMS and answering it takes a lot of time. Honestly, I don't think I could answer patients contacting me through an app." (50)

**Source paper**	**Mood monitoring is confronting**	**Burden of mood monitoring**	**Compulsive monitoring**	**Decrease motivation**	**Unwelcome reminder of mental illness**
Bos et al 2019	x	x			
de Angel et al 2022	x				x
de Angel et al 2023	x	x	x	x	x
Drake et al 2012	x	x		x	x
Folkersma et al 2021	x				x
Hetrick et al 2018	x	x			
Incecik et al 2020	x	x	x		
Meng et al 2018	x				
Patoz et al 2021	x	x			
Simblett et al 2020	x				
Simblett et al 2024					
Thomson et al 2024					
Van Tiem et al 2021					
White et al 2023	x	x		x	

Supplementary Table 4: sub-themes of negative psychological effects of mood monitoring

Source paper	No subsequent behaviour change	No additional benefit	Participants consider it effective
Bos et al 2019	x	x	
de Angel et al 2022			x
de Angel et al 2023			x
Drake et al 2012		x	x
Folkersma et al 2021		x	x
Hetrick et al 2018			
Incecik et al 2020	x	x	x
Meng et al 2018			
Patoz et al 2021			
Simblett et al 2020			
Simblett et al 2024			
Thomson et al 2024			x
Van Tiem et al 2021		x	
White et al 2023			

Supplementary Table 5: perceived effectiveness

Source paper	Regular use is important	Difficult to track mood when unwell	Difficulty answering and interpreting the limited active measures	Active ambulatory assessment repetitive
Bos et al 2019				x
de Angel et al 2022				x
de Angel et al 2023				x
Drake et al 2012	x			x
Folkersma et al 2021				x
Hetrick et al 2018				
Incecik et al 2020		x	x	x
Meng et al 2018		x		
Patoz et al 2021		x	x	
Simblett et al 2020				
Simblett et al 2024				

Thomson et al 2024				
Van Tiem et al 2021				
White et al 2023		x	x	x
**Supplementary Table 6: difficulties in completing questionnaires**				

**Source paper**	**Direct access to a clinician through the app**	**Sharing with clinician improves understanding and communication**	**Sharing with family/friends/people with depression creates community and provides support**	**Sharing depends on trust**	**Clinician is not interested in the information**	**Clinician could misinterpret data**	**Sharing with clinician feels invasive**	**Don't want to medicalise other relationships**	**Don't want to burden others**	**Concerned about replacement of human contact by digital app**
Bos et al 2019		x		x			x			x
de Angel et al 2022		x	x	x						x
de Angel et al 2023		x								x
Drake et al 2012							x		x	
Folkersma et al 2021										
Hetrick et al 2018										
Incecik et al 2020	x									x
Meng et al 2018				x						
Patoz et al 2021	x									x
Simblett et al 2020										
Simblett et al 2024										
Thomson et al 2024	x									
Van Tiem et al 2021						x				
White et al 2023		x		x	x	x	x			
**Supplementary Table 7: sharing with others**										

**Source paper**	**Other aspects in addition to mood**	**Concerns around price and**	**Crisis/wellness plan or procedure**	**Ease of use**	**Graphical display of mood**	**Reminder notifications to complete**	**Support required prior to/du**	**Personalisation**	**Positive messages**	**Preference for the wearable device**	**Preference for smartphone app over**	**Preference for digital methods**	**Full control over many aspects of the**	**Effectiveness/ scientific evaluation**

	**monito ring**	**accessi bility**				**ambula tory assess ment**	**ring use**			**over smartp hone app**	**wearab le device**		**proces s/app data**	
Bos et al 2019					x			x					x	x
de Angel et al 2022	x	x					x	x		x	x	x	x	x
de Angel et al 2023	x			x				x						
Drake et al 2012														
Folker sma et al 2021								x						
Hetric k et al 2018	x		x					x						
Inceci k et al 2020				x		x		x						
Meng et al 2018							x	x	x				x	
Patoz et al 2021	x	x	x	x			x	x						
Simbl ett et al 2020	x								x					
Simbl ett et al 2024			x		x			x	x				x	
Thom son et al 2024	x				x			x	x			x		
Van Tiem et al 2021														
White et al 2023				x	x		x	x		x			x	

Supplementary Table 8a: sub-themes of desired features of app

**Source paper**	**Advantages of passive ambulatory assessment**	**Wearables comfortable**	**App feedback to not be patronising**	**Address technical issues**	**Address data security concerns**	**Easily interpretable data/results**
Bos et al 2019	x					
de Angel et al 2022	x				x	x
de Angel et al 2023		x		x		

Drake et al 2012			x			
Folkersma et al 2021						
Hetrick et al 2018						
Incecik et al 2020				x		x
Meng et al 2018	x				x	x
Patoz et al 2021					x	x
Simblett et al 2020						
Simblett et al 2024						
Thomson et al 2024	x					
Van Tiem et al 2021						
White et al 2023	x	x		x		x
**Supplementary Table 8b: sub-themes of desired features of app**						

Source paper	Provide accountability/ goal setting	Aid/monitor treatment	Improve insight	Memory aid e.g. medication adherence	Monitor sleep/physical health	Improve motivation	Objectively judge mood	Reassurance	Relapse prevention	Improves self-management
Bos et al 2019		**x**	**x**				**x**		**x**	**x**
de Angel et al 2022		**x**	**x**	**x**	**x**	**x**	**x**			
de Angel et al 2023	x	x	x		x	x				
Drake et al 2012			x			x	x			
Folkersma et al 2021	x	x	x			x	x			
Hetrick et al 2018		x								
Incecik et al 2020			x				x	x		
Meng et al 2018	x	x	x				x			
Patoz et al 2021		x	x				x	x		
Simblett et al 2020		x	x		x		x		x	
Simblett et al 2024			x		x		x		x	x
Thomson et al 2024	x	x	x		x		x			x
Van Tiem et al 2021			x			x				x
White et al 2023		x	x		x	x	x	x	x	x

Supplementary Table 9: sub-themes of purpose of app

Source paper	Facilitator: monitor treatment adherence	Facilitator: improves clinical decision making	Facilitator: improve efficiency of time with clinician	Facilitator: positive reinforcement/feedback	Facilitator: overcome subjective judgement of mood	Barrier: decreases autonomy / managing expectations	Barrier: difficulty in integrating into practice	Barrier: increased workload	Barrier: difficult to interpret results	Barrier: concern about risk/liability issues

Bos et al 2019							x			
de Angel et al 2022		x				x		x	x	x
de Angel et al 2023									x	
Drake et al 2012										
Folkersma et al 2021									x	
Hetrick et al 2018						x		x	x	x
Incecik et al 2020			x							
Meng et al 2018	x	x		x				x	x	x
Patoz et al 2021	x	x			x		x	x		x
Simblett et al 2020	x									
Simblett et al 2024										
Thomson et al 2024										
Van Tiem et al 2021	x									
White et al 2023							x	x		

Supplementary Table 10: clinician barriers and clinician facilitators

Supplementary Information 3: Systematic review search strategy & PRISMA flow diagram

Search performed 3/3/23. The search strategy was trialled on one database first and then refined subsequently. The search results were uploaded to Rayyan (67). Search terms were determined based on discussion between researchers, previous reviews and consultation with specialist librarians. The search was performed from inception to 3/3/23. The search was updated on 28/10/24.

Number of abstracts original search 3/3/23:
Medline: 2984
Embase: 4827
PsychINFO: 3346
SCOPUS: 2321
IEE Xplore: 615
Proquest dissertations and theses global: 2697
Proquest SciTech Collection: 3489
Total: 20,279
Full text review: 565

Number of abstracts of updated search 3/3/24 – 28/10/24:
Medline: 494
Embase: 920
PsychINFO: 364
SCOPUS: 1088
IEE Xplore: 99
Proquest dissertations and theses global: 0
Proquest SciTech Collection: 469
Total prior to deduplication: 3236
Auto-deduplicated: 1119
Total: 2117
Full text review: 193

Published literature:

**OVID Medline**	
1	exp bipolar disorder/ OR exp depression OR exp mania/
2	(((bipolar or bi polar) adj5 (disorder$ or depress$)) or ((cyclothymi$ or rapid or ultradian) adj5 cycl$) or hypomani$ or mania$ or manic$ or mixed episode$ or rcbd).mp
3	('Depressive Disorder' OR 'Depression' OR 'dysthymi*' OR 'affective disorder' OR 'affective disorders' OR 'mood disorder' OR 'mood disorders' OR 'depression*' OR 'depressive*' OR 'dysthymic disorder').mp
4	1 OR 2 OR 3
5	('self monitor*' or 'self assess*' or 'self manag*' or 'self record*' or 'self surveillance' or 'patient* monitor*' or 'measurement technolog*' or 'telemonitor*' or 'remote monitor*' or 'passive monitor*' or 'active monitor*' or 'mood track*' or 'mood monitor*' or 'experience sampl*' or 'ecological momentary assessment').mp
6	4 adj10 5

https://ovidsp.ovid.com/ovidweb.cgi?T=JS&NEWS=N&PAGE=main&SHAREDSEARCHID=10Q4IjupCc3HoHvVXInK959r2tcDy9vTlJlQsifUVfSVSyNrczwzGqVCqql3svtpo

**OVID EMBASE**	
1	bipolar disorder/ or bipolar depression/ or bipolar I disorder/
2	depression assessment/ or treatment resistant depression/ or minor depression/ or chronic depression/ or postnatal depression/ or atypical depression/ or antenatal depression/ or

	adolescent depression/ or "mixed mania and depression"/ or post-stroke depression/ or endogenous depression/ or major depression/ or recurrent brief depression/ or depression/ or bipolar depression/ or perinatal depression/ or agitated depression/ or organic depression/
3	"mixed mania and depression"/ or mania/ or bipolar mania/
4	(((bipolar or bi polar) adj5 (disorder$ or depress$)) or ((cyclothymi$ or rapid or ultradian) adj5 cycl$) or hypomani$ or mania$ or manic$ or mixed episode$ or rcbd).mp.
5	('Depressive Disorder' or 'Depression' or 'dysthymi*' or 'affective disorder' or 'affective disorders' or 'mood disorder' or 'mood disorders' or 'depression*' or 'depressive*' or 'dysthymic disorder').mp.
6	1 OR 2 OR 3 OR 4 OR 5
7	('self monitor*' or 'self assess*' or 'self manag*' or 'self record*' or 'self surveillance' or 'patient* monitor*' or 'measurement technolog*' or 'telemonitor*' or 'remote monitor*' or 'passive monitor*' or 'active monitor*' or 'mood track*' or 'mood monitor*' or 'experience sampl*' or 'ecological momentary assessment').mp.
8	6 adj10 7

https://ovidsp.ovid.com/ovidweb.cgi?T=JS&NEWS=N&PAGE=main&SHAREDSEARCHID=2Ofrc9VijRp6L40USOJFeEn3I1bHOmCW8O1Hzfz13xklneo3jW1767QyyDQMHnVDj

**OVID PsychINFO**	
1	Bipolar Disorder/ or Bipolar II Disorder/ or Bipolar I Disorder/ or Mania/
2	Major Depression/ or Endogenous Depression/ or Postpartum Depression/ or Recurrent Depression/ or "Depression (Emotion)"/ or Reactive Depression/ or Late Life Depression/ or Atypical Depression/ or Treatment Resistant Depression/ or "Long-term Depression (Neuronal)".mp.
3	(((bipolar or bi polar) adj5 (disorder$ or depress$)) or ((cyclothymi$ or rapid or ultradian) adj5 cycl$) or hypomani$ or mania$ or manic$ or mixed episode$ or rcbd).mp
4	'Depressive Disorder' OR 'Depression' OR 'dysthymi*' OR 'affective disorder' OR 'affective disorders' OR 'mood disorder' OR 'mood disorders' OR 'depression*' OR 'depressive*' OR 'dysthymic disorder'
5	1 OR 2 OR 3 OR 4
6	('self monitor*' or 'self assess*' or 'self manag*' or 'self record*' or 'self surveillance' or 'patient* monitor*' or 'measurement technolog*' or 'telemonitor*' or 'remote monitor*' or 'passive monitor*' or 'active monitor*' or 'mood track*' or 'mood monitor*' or 'experience sampl*' or 'ecological momentary assessment')
7	5 adj10 6

https://ovidsp.ovid.com/ovidweb.cgi?T=JS&NEWS=N&PAGE=main&SHAREDSEARCHID=7WPhKe8RR9Athylx2jCCPdAkgbQlgcrdVpxl6NGPhskh73E8wr3X16vfACAP9Q54Y

SCOPUS:
TITLE-ABS-KEY((68) OR (69) OR {self-assess*} OR {self manag*} OR {self-manag*} OR {self record*} OR {self-record*} OR {self surveillance} OR {self-surveillance} OR {patient* monitor*} OR {patient*-monitor*} OR {measurement technolog*} OR {measurement-technolog*} OR {telemonitor*} OR {remote monitor*} OR {remote-monitor*} OR {passive monitor*} OR {passive-monitor*} OR {active monitor*} OR {active-monitor*} OR {mood track*} OR {mood-track*} OR {mood monitor*} OR {mood-monitor*} OR {experience sampl*} OR {ecological momentary assessment}) W/10 ({Bipolar disorder*} OR {Manic depress*} OR {Manic-depress*} OR {Bipolar affective psychos*} OR {Bipolar depress*} OR {Manic disorder*} OR (70) OR {depressive disorder*} OR {major depressive disorder*} OR (70) OR {affective disorder*} OR {mood disorder*})

IEE XPLORE:
('self monitor' OR 'self monitoring' OR 'self assess' OR 'self assessment' OR 'self manage' OR 'self management' OR 'self record' OR 'self recording' OR 'self surveillance' OR 'patient monitor' OR

'patient monitoring' OR 'measurement technology' OR 'telemonitor' OR 'telemonitoring' OR 'remote monitor' OR 'remote monitoring' OR 'passive monitor' OR 'passive monitoring' OR 'active monitor*' OR 'mood track*' OR 'mood monitor*' OR 'experience sample' OR 'experience sampling' OR 'ecological momentary assessment') NEAR/10 ('Bipolar*' OR 'Manic disorder*' OR 'depressi*' OR 'affective disorder*' OR 'mood disorder*')

Google scholar search: An additional search of the first 15 pages of Google Scholar was conducted (search terms: 'mood track', 'ecological momentary assessment', 'monitoring', 'remote monitoring', 'active monitor', 'passive monitor', 'experience sample', 'experience sampling')

Finally, subject experts were approached to identify additional articles.

Grey Literature:

ProQuest Dissertations & Theses Global:
(("self monitor" OR "self monitoring" OR "self monitors") OR ("self assess" OR "self assessed" OR "self assessment") OR ("self manage" OR "self managed" OR "self managing") OR ("self record" OR "self recorded" OR "self recording") OR "self surveillance" OR "patient* monitor*" OR ("measurement technologies" OR "measurement technology") OR "telemonitor*" OR ("remote monitoring") OR ("passive monitoring") OR ("active monitoring") OR "mood track*" OR "mood monitor*" OR ("experience sampling") OR "ecological momentary assessment") NEAR/10 (("bipolar disorder" OR "bipolar disorders") OR ("manic depression" OR "manic depressive") OR "Bipolar affective psychos*" OR ("bipolar depression") OR "Manic disorder*" OR "depressi*" OR ("depressive disorder") OR "major depressive disorder*" OR "depression" OR ("affective disorder" OR "affective disorders") OR ("mood disorder" OR "mood disorders"))

http://abc.cardiff.ac.uk/login?url=https://www.proquest.com/search/2332884?accountid=9883
https://www.proquest.com/pqdtglobal

ProQuest SciTech Premium Collection:

(("self monitor" OR "self monitoring" OR "self monitors") OR ("self assess" OR "self assessed" OR "self assessment") OR ("self manage" OR "self managed" OR "self managing") OR ("self record" OR "self recorded" OR "self recording") OR "self surveillance" OR "patient* monitor*" OR ("measurement technologies" OR "measurement technology") OR "telemonitor*" OR ("remote monitoring") OR ("passive monitoring") OR ("active monitoring") OR "mood track*" OR "mood monitor*" OR ("experience sampling") OR "ecological momentary assessment") NEAR/10 (("bipolar disorder" OR "bipolar disorders") OR ("manic depression" OR "manic depressive") OR "Bipolar affective psychos*" OR ("bipolar depression") OR "Manic disorder*" OR "depressi*" OR ("depressive disorder") OR "major depressive disorder*" OR "depression" OR ("affective disorder" OR "affective disorders") OR ("mood disorder" OR "mood disorders"))

http://nottingham.idm.oclc.org/login?url=https://www.proquest.com/search/2332894?accountid=8018
https://www.proquest.com/scitechpremium/

Google incognito mode – first 200 results:
No new papers identified

Systematic Reviews reference checked:
No new papers identified

Supplementary Figure 1: PRISMA flow diagram of included studies

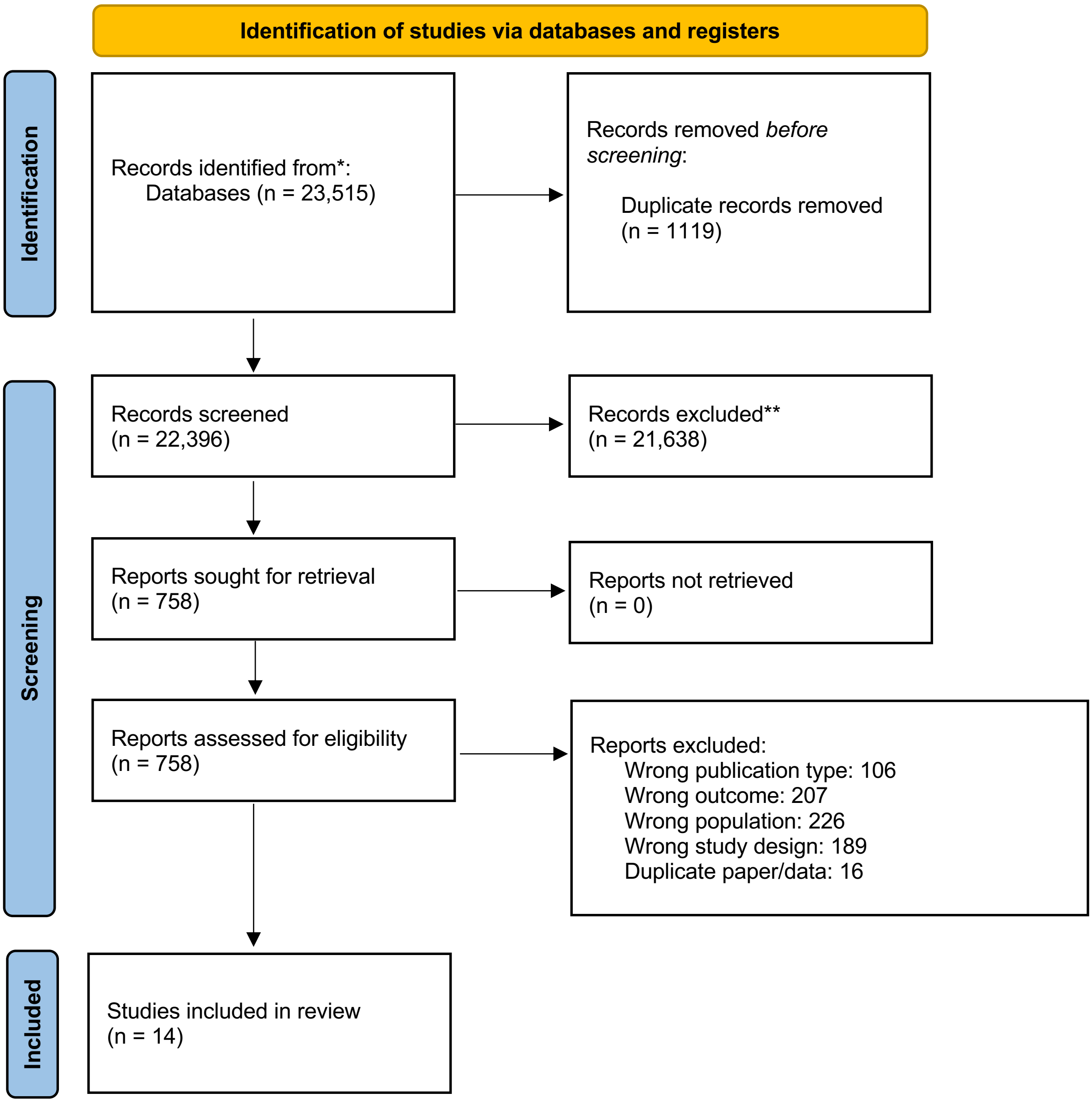

PRISMA Checklist

Section/topic	#	Checklist item	Reported on page #
**TITLE**			
Title	1	Identify the report as a systematic review, meta-analysis, or both.	1
**ABSTRACT**			
Structured summary	2	Provide a structured summary including, as applicable: background; objectives; data sources; study eligibility criteria, participants, and interventions; study appraisal and synthesis methods; results; limitations; conclusions and implications of key findings; systematic review registration number.	1
**INTRODUCTION**			
Rationale	3	Describe the rationale for the review in the context of what is already known.	2
Objectives	4	Provide an explicit statement of questions being addressed with reference to participants, interventions, comparisons, outcomes, and study design (PICOS).	2
**METHODS**			
Protocol and registration	5	Indicate if a review protocol exists, if and where it can be accessed (e.g., Web address), and, if available, provide registration information including registration number.	18
Eligibility criteria	6	Specify study characteristics (e.g., PICOS, length of follow-up) and report characteristics (e.g., years considered, language, publication status) used as criteria for eligibility, giving rationale.	18

Information sources	7	Describe all information sources (e.g., databases with dates of coverage, contact with study authors to identify additional studies) in the search and date last searched.	18, Supplementary materials
Search	8	Present full electronic search strategy for at least one database, including any limits used, such that it could be repeated.	Supplementary materials
Study selection	9	State the process for selecting studies (i.e., screening, eligibility, included in systematic review, and, if applicable, included in the meta-analysis).	Supplementary materials
Data collection process	10	Describe method of data extraction from reports (e.g., piloted forms, independently, in duplicate) and any processes for obtaining and confirming data from investigators.	18
Data items	11	List and define all variables for which data were sought (e.g., PICOS, funding sources) and any assumptions and simplifications made.	18
Risk of bias in individual studies	12	Describe methods used for assessing risk of bias of individual studies (including specification of whether this was done at the study or outcome level), and how this information is to be used in any data synthesis.	18
Summary measures	13	State the principal summary measures (e.g., risk ratio, difference in means).	18
Synthesis of results	14	Describe the methods of handling data and combining results of studies, if done, including measures of consistency (e.g., I^2) for each meta-analysis.	18
